# Class C CpG Oligodeoxynucleotide Immunomodulatory Response in Aged Squirrel Monkey (*Saimiri Boliviensis Boliviensis*)

**DOI:** 10.3389/fnagi.2020.00036

**Published:** 2020-03-03

**Authors:** Pramod N. Nehete, Lawrence E. Williams, Sriram Chitta, Bharti P. Nehete, Akash G. Patel, Margish D. Ramani, Thomas Wisniewski, Henrieta Scholtzova

**Affiliations:** ^1^Department of Comparative Medicine, The University of Texas MD Anderson Cancer Center, Bastrop, TX, United States; ^2^The University of Texas Graduate School of Biomedical Sciences, Houston, TX, United States; ^3^Department of Neurology, Center for Cognitive Neurology, New York University School of Medicine, New York, NY, United States; ^4^Department of Pathology, New York University School of Medicine, New York, NY, United States; ^5^Department of Psychiatry, New York University School of Medicine, New York, NY, United States

**Keywords:** CpG ODN class C, aged squirrel monkey, cerebral amyloid angiopathy, cellular immune response, ELISPOT, cytokines

## Abstract

One means of stimulating the mammalian innate immune system is *via* Toll-like receptor 9 (TLR9) being exposed to unmethylated cytosine-phosphate-guanine (CpG) DNA, also known as pathogen-associated molecular patterns (PAMPs) of microbial origin. Synthetic CpG oligodeoxynucleotides (ODNs) with defined CpG motifs possess broad immunostimulatory properties that make CpG ODNs suitable as therapeutic interventions in a variety of human disease conditions, including Alzheimer’s disease (AD). Rodent models are often used to preclinically test the effectiveness of CpG ODN therapeutic agents for AD and other disorders. However, the translatability of findings in such models is limited due to the significant difference of the expression of TLR9 between primates and rodents. The squirrel monkey (SQM), a New World non-human primate (NHP), is known to be phylogenetically proximate to humans, and develops extensive age-dependent cerebral amyloid angiopathy (CAA), a key pathological feature of AD. Hence, this model is currently being used to test AD therapeutics. In the present study, we conducted the first examination of Class C CpG ODN’s immunomodulatory role in elderly SQMs. We documented the effectiveness of CpG ODN to trigger an immune response in an aged cohort whose immune system is senescent. The specific immune response patterns detected here closely resembled CpG ODN-induced immunostimulatory patterns observed in prior human studies. Overall, our findings provide critical data regarding the immunomodulatory potential of CpG ODN in this NHP model, allowing for future translational studies of innate immunity stimulation *via* TLR9 agonists for diverse indications, including AD therapeutics.

## Introduction

The mammalian innate immune system naturally recognizes unmethylated cytosine-phosphate-guanine (CpG) DNA as pathogen-associated molecular patterns (PAMPs) indicative of viruses or bacteria. CpG oligodeoxynucleotides (ODN), as synthetic ODNs with defined CpG motifs, mimic the structure of these pathogenic DNA to incite an innate immune response. The immunomodulatory properties of CpG ODN are enacted through an endosomal pattern recognition receptor (PRR), the Toll-like receptor 9 (TLR9), to propagate a specific downstream innate immune response (Krieg, 2003, 2006, 2012).

Three classes of TLR9 agonists, CpG ODN Class A, B, and C, each pose a distinct biologic activity, due to their unique structures and different cell activations, to produce varying innate immune signaling cascades. Class A CpG ODNs are single CpG motifs of partially phosphorothioated (PS-modified) and phosphodiester backbone bases in a palindromic sequence that induce the production of IFNα by peripheral dendritic cells (pDCs) and indirectly activate NK cells. Class B CpG ODNs are multiple CpG motifs composed of fully PS-modified nucleotides. Unlike Class A CpG ODNs, Class B contain B-cell activators and stimulate pDC maturation. The Class C CpG ODNs, which combine characteristics of the A and B classes, are generally fully PS-modified, double-stranded palindromic motifs that induce strong IFNα production, pDC maturation, and efficient B cell stimulation (Scheiermann and Klinman, 2014; Adamus and Kortylewski, 2018).

Data from studies in mice and ongoing human clinical trials suggest that CpG ODNs are effective and safe vaccine adjuvants, and provide support for the development of CpG ODNs as potential therapeutic agents for allergic, neoplastic diseases (Dorn and Kippenberger, 2008; Vollmer and Krieg, 2009; Brody et al., 2010), and Alzheimer’s disease (AD; Crack and Bray, 2007; Brody and Holtzman, 2008; Scholtzova et al., 2009, 2014, 2017; Drummond et al., 2018; Wisniewski and Drummond, 2019). Although CpG ODN is known to be a potent immunostimulant in mice, the immunomodulatory effects are not as pronounced in larger mammals, including humans. The difference in activation is due to TLR9 expression being abundant across macrophage and myeloid dendritic cells (mDCs) in rodents, while being limited to plasmacytoid dendritic cells (pDCs) and B cells in humans and non-human primates (NHP; Krieg, 2006, 2012; Scheiermann and Klinman, 2014). Hence, despite the promising immunomodulatory capabilities of CpG ODN in mice, further characterization of its effects in NHPs may better define its effects in humans in terms of safety and therapeutic effect for various indications (Verthelyi and Klinman, 2003; Klinman et al., 2010).

Squirrel monkeys (SQM), small New World NHP known to be phylogenetically proximate to humans, have served an important role in studying pathogenesis of human disease conditions such as AD (Elfenbein et al., 2007; Heuer et al., 2012, 2017; Rosen et al., 2016), malaria (Collins et al., 2009), HIV (LaBonte et al., 2002), and Creutzfeldt-Jakob disease (Williams et al., 2007; Ritchie et al., 2016), as well as in the development of therapeutic strategies (Tougan et al., 2013). We intend to utilize this NHP model to advance studies conducted in mice and human clinical trials for further characterizing CpG ODN immunostimulatory profiles so as to reach efficient translation for human benefit (Scholtzova et al., 2009, 2014, 2017; Riccio et al., 2015).

To the best of our knowledge, this is the first documentation describing the CpG ODN immunomodulatory role in elderly SQMs by directing efforts towards deciphering lymphocyte and monocyte subset surface antigen expressions and functions. Our present findings also represent the first report identifying pDC markers in SQMs. We took advantage of an ongoing, long-term Class C CpG ODN treatment on aged female SQMs being studied as an NHP model of sporadic cerebral amyloid angiopathy (CAA).

## Materials and Methods

### Monkey, Care and Housing

Subject animals consisted of elderly female SQMs (*Saimiri boliviensis boliviensis)*. They were selected from our ongoing AD study using SQMs as a model of naturally occurring brain vascular amyloid pathology (at the SQM Breeding Research Resources (SMBRR) located at the University of Texas MD Anderson Cancer Center Michale E. Keeling Center for Comparative Medicine and Research). These monkeys were housed in social groups within two connecting cages that were 4′ wide × 6′ tall × 14′ long.

### Diet

Animals had *ad libitum* access to New World Primate Diet (Purina #5040) and water. In addition, they were fed either fresh fruit or vegetables daily. Specialty foods, such as seeds, peanuts, raisins, yogurt, cereals, frozen juice cups and peanut butter, were distributed daily to them as enrichment. At no time were the subjects ever food- or water-deprived. Subjects were also provided with destructible enrichment manipulanda and different travel/perching materials on a rotating basis to promote the occurrence of typical species behavior.

### Study Groups

The study population consisted of aged female SQMs (18–20 years of age). A subset of 10 CpG ODN-injected monkeys was selected for our analyses. All subjects were considered healthy and in their normal social groups at the time they were sampled. A SQM’s average life span is 20 years and maximum life expectancy of <25 years (Abee, 2000; Williams and Glasgow, 2000; Williams, 2008). With an estimated maximum life-span of 122 years in humans (Lucke et al., 2010; Pignolo, 2019) the rate of aging in SQMs is roughly 3.4 times as fast. Thus, SQMs offer a distinct advantage over long-term human aging and Alzheimer’s research.

### CpG ODN

Endotoxin-free Class C CpG ODN, a 30-mer phosphothioate ODN containing juxtaposed CpG motifs with flanking regions in a self-complimentary palindromic sequence (5′-TCGAACGTTCGAACGTTCGAACGTTCGAAT-3′), was purchased from Integrated DNA Technologies, Inc.

### Blood Collection and CpG ODN Administration

Blood samples (2–3 ml) were collected in EDTA coated collection tubes from the femoral vein of study animals which had been injected twice with Class C CpG ODN (2 mg/kg) at two intervals, four to 5 weeks apart. CpG ODN was administered subcutaneously (s.c.) into the interclavicular space. All injections and blood sample collections (day 0/baseline, day 1, day 3, and day 7 post-CpG ODN) occurred in the morning (8–9 AM) before the animals were fed.

### Hematologic Analyses and PBMC Preparation

Hematology was performed on EDTA-preserved blood by using an automated analyzer Advia (Siemens Healthcare Diagnostics, Tarrytown, NY, USA). The absolute number of lymphocytes and monocytes, as obtained from hematologic analysis, was used to convert the frequency of the lymphocyte population obtained from FACS analysis to calculate the absolute numbers in each of the lymphocyte and monocyte subset populations. The assessment of CpG ODN immunostimulatory activity was first examined in whole blood samples collected prior to injection (baseline/day 0) and on day 1, day 3, and day 7 after CpG ODN administration.

Blood samples collected from SQMs prior to CpG ODN injection were used for peripheral blood mononuclear cells (PBMCs) isolation. PBMCs were isolated by Ficoll-Hypaque density gradient separation as described previously (Nehete et al., 2003, 2005, 2013, 2018). Erythrocytes were removed by osmotic lysis in ACK lysing buffer (Life Technologies, Grand Island, NY, USA), and the remaining viable cells were counted on Cellometer (Nexcelon Bioscience, LLC, Lawrence, MA, USA) and used for various immune assay. PBMCs were used in our subsequent *in vitro* analyses, including viability and functional assays, and flow cytometry characterization of CpG ODN effects on expression of major lymphocyte and monocyte subsets.

### Flow Cytometry

A series of commercially available human monoclonal antibodies were tested for cross reactivity to *Saimiri boliviensis boliviensis* mononuclear cells using flow cytometric analysis. Mouse anti-human monoclonal antibodies against CD3 (Clone SP34–2), CD16 (Clone 3G8), CD14 (Clone M5E2), CD20 (Clone L27), CD123 (Clone 7G3), HLA DR (Clone L243), and appropriate isotype-control antibodies which had been conjugated to either fluoroscein isothiocyanate (FITC), phycoerythrin (PE), peridinin-chlorophyl-protein (PerCP), PE-Cy5, or allophycocyanin (APC), were used for these studies (Becton Dickinson-Pharmingen, San Jose, CA, USA). The Lin1-FITC antibody cocktail containing anti-CD3, CD14, CD16, CD20, as well as antibodies against CD123 marker (PE) and HLA DR (PerCP) were used as part of the identification of plasmacytoid DCs (pDCs; Becton Dickinson-Pharmingen). Phenotypic characterization of lymphocytes and monocytes in peripheral blood from the monkeys was performed by cell surface staining of whole blood samples and flow cytometry analysis as described previously (Nehete et al., 2013, 2018). Briefly, 100 μl of EDTA-preserved whole blood from each sample was added to individual 12 mm × 75 mm polystyrene test tubes (Falcon, Lincoln Park, NJ, USA) containing pre-added monoclonal antibodies against CD3, CD14, CD16, CD20 (Lin-FITC cocktail), HLA DR (PerCP), and CD123 (PE), and incubated for 15 min at room temperature in the dark. After removing the red blood cells by incubating with FACS lysing solution (Becton Dickinson, Franklin Lakes, NJ, USA), the mononuclear cells were washed twice with phosphate-buffered saline (PBS) and re-suspended in 1% paraformaldehyde solution. The stained cells were acquired on a FACS Celesta™ (Becton Dickinson, CA, USA). All samples evaluated in this study were compensated for spectral overlap of one fluorochrome into other fluorochrome using singly-fluorochrome labeled cells. All antibodies used in this study are cross reactive to *Saimiri boliviensis* *boliviensis*, as reported previously (Nehete et al., 2013, 2018) and in NIH Nonhuman Primate Reagent Resource^1^. In this study, we described two types of monocyte population, the classical monocytes expressing CD14+ cell surface receptor (CD14+ CD16− monocytes), and the non-classical monocytes co-expressing CD14+ and CD16+ receptors (CD14+ CD16+ monocytes).

To evaluate CD123 expression on T cells, B cells, and monocytes, PBMCs were stimulated with CpG ODN for 30 h at 37°C. PBMCs were harvested and washed with cold PBS without Ca^2+^ or Mg^2+^. Cells that were positive for experimental markers had mean channel fluorescence values ranging from 10- to more than 20-fold higher. PBMCs were then washed, incubated with appropriate antibodies for 20 min, washed, and fixed with 2% formaldehyde and stored at 4°C for analysis. The samples were acquired on a FACS Celesta™ flow cytometer using DIVA software (BD) and analyzed using FlowJO (Tri Star, CA, USA). Logical gating was used to identify specific innate immune cell populations (pDCs, B cells, T cells, and monocytes) and the activation or maturation markers expressed by these cells are shown as a percentage of the parent population.

### Viability by MTT Assay

A proliferation dye reduction assay was used to determine a viability of PBMCs. PBMCs were cultured in triplicate at 1 × 10^5^/well in 96-well round-bottomed plates (Becton Dickinson, Franklin Lakes, NJ, USA) with 5 and 50 μg/ml Class C CpG ODN or 5 μg/ml concanavalin A (Con A) in a volume of 200 μl. Cultures were incubated in a humidified 5% CO_2_ environment at 37°C for 96 h. At the end of the culture period, supernatants were harvested and cells were assayed for viability content by the standard MTT dye reduction assay described previously (Nehete et al., 2017a,b,c, 2018).

### ELISPOT Assay for Detecting Antigen-Specific IFNγ and IL12p40 Producing Cells

Freshly-isolated PBMC, as described above, were stimulated with the mitogens CpG ODN (5 μg/ml and 50 μg/ml final concentrations) and 5 μg/ml of Con A to determine the numbers of IFNγ- or IL12p40-producing cells by the Enzyme Linked Immuno Spot (ELISPOT) assay using the methodology reported earlier (Nehete et al., 2003, 2005). Briefly, aliquots of PBMC (10^5^/well) were seeded in triplicate wells of 96-well plates (polyvinylidene difluoride backed plates, MAIP S 45, Millipore, Bedford, MA, USA) pre-coated with the primary IFNγ or IL12p40 antibody, and the lymphocyte were stimulated with the different mitogens or antigen. After incubation for 30–32 h at 37°C, the cells were removed, and the wells were thoroughly washed with PBS and developed as per the protocol provided by the manufacturer. Purple colored spots representing individual cells secreting IFNγ or IL12p40 were counted by an independent agency (Zellnet Consulting, New Jersey, NJ, USA) using the KS-ELISPOT automatic system (Carl Zeiss, Inc. Thornwood, NY, USA) for the quantitative analysis of the number of IFNγ or IL12p40 spot forming cells (SFC) for 10^5^ input PBMC. Responses were considered positive when the number of SFCs with the test antigen was at least five and was five above the background control values from cells cultured in the medium alone.

### IFNα Luminex Magnetic Bead Assay

IFNα cytokine was measured in cell-free PBMC supernatant using MILLIPLEX-_MAP_ human cytokine/chemokine magnetic bead panel (EMD Millipore Corporation, Billerica, MA, USA) according to the manufacturers’ protocols. There is 91.4%–98.1% homology between the nucleotide sequences of SQM cytokine genes and published sequences of equivalent human and nonhuman primate genes (Heraud et al., 2002). Briefly, supernatant samples were centrifuged (14,000× *g* for 5 min) and 25 μl of aliquots were used in assay. The 96-well filter plate was blocked with assay buffer for 10 min at room temperature, washed, and 25 μl of standard or control samples were dispersed into appropriate wells. After adding 25 μl of beads to each well, the plate was incubated on a shaker overnight at 4°C. The next day, after washing two times with wash buffer, the plate was incubated with detection antibody for 1 h at room temperature and again incubated with 25 μl of Streptavidin-Phycoerythin for 30 min at room temperature. After washing two times with wash buffer, 150 μl of sheath fluid was added into each well and multianalyte profiling was performed on the Bio-Plex 200 system (Luminex X MAP technology). Calibration microspheres for classification and reporter readings as well as sheath fluid, assay, and wash buffer were also purchased from Bio-Rad (Hercules, CA, USA). Acquired fluorescence data were analyzed by the Bio-Plex manager 5.0 (Bio-Rad, Hercules, CA, USA). All steps of incubations were performed on a shaker. The minimum detectable concentration was calculated by the Multiplex Analyst immunoassay analysis Software from Millipore. The minimum IFNα detectable concentration was 2.9 pg/ml.

### Statistical Analysis

**Figure 1:**
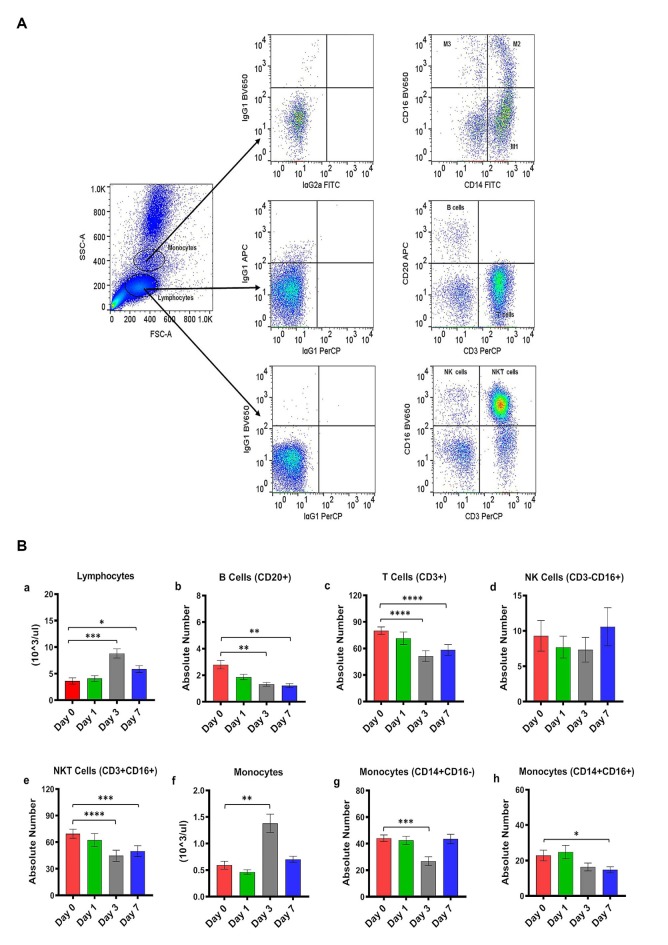
**(A)** Gating scheme for identification of the various cell markers in the peripheral blood. The lymphocytes and monocytes were first gated based on forward scatter (FSC) vs. side scatter (SSC). CD3+ and CD20+ cells were positively identified by the lymphocyte gate while CD16+ and CD14+ cells were positively identified by the monocyte gate. The specificity of staining for the various markers was ascertained according to the isotype control antibody staining used for each pair of combination markers, as shown. **(B)** Changes in lymphocytes and monocytes populations in CpG ODN stimulated SQM peripheral blood across 7 days. Hematology analysis of lymphocyte (**a**) and monocytes (**f**) was performed on EDTA-preserved whole blood by using an automated analyzer, Advia (Siemens Healthcare Diagnostics, Tarrytown, NY, USA). Values on the Y-axis are the absolute numbers of lymphocytes and monocytes presented as 10^3^ per μl of whole blood. Aliquots of the whole blood were stained with fluorescence-labeled antibodies to B cells (CD20+; **b**), T cells (CD3+; **c**), NK cells (CD3-CD16+; **d**), and NKT cell (CD3 + CD16+; **e**) to identify lymphocyte subpopulations, and stained with antibodies to identify monocyte subpopulations (CD14+CD16−; **g**) and (CD14+CD16+; **h**) at day 0, 1, 3, and 7 by flow cytometry. Values on the Y-axis are the absolute numbers of lymphocytes or monocytes. ****p < 0.0001; ***p < 0.001; **p < 0.01; *p < 0.05; ns, *p* > 0.05.

**Figure 2:**
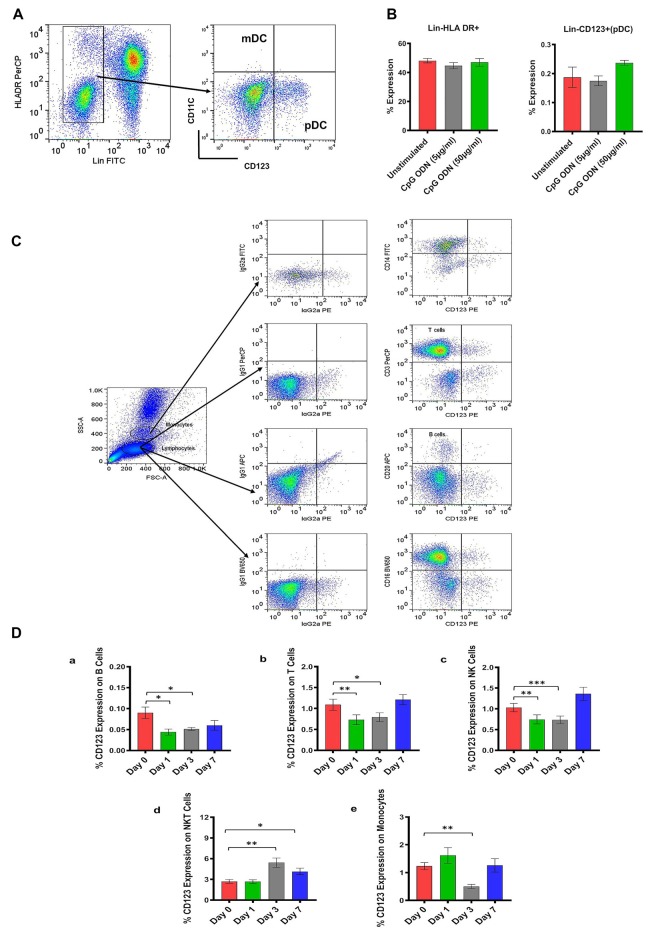
**(A)** Gating scheme for identification of pDC (Lin-HLA DR+CD123+) in the peripheral blood. **(B)** Characterization of SQM plasmacytoid dendritic cells (pDCs) and analysis of CpG ODN effect on Lin-HLA DR+ and Lin-CD123+ expressing cells. Aliquots of the whole blood were stained with the Lin1-FITC antibody cocktail consisting of FITC-labeled antibodies to CD3, CD14, CD16, CD20, PE-labeled antibodies to CD123, and PerCP-labeled antibodies to HLA DR to identify pDCs. Values on the Y-axis represent % expression of Lin1-HLA DR+ and Lin-CD123+ cells evaluated in the presence (5 μg/ml and 50 μg/ml) or absence (unstimulated) of CpG ODN. *P*-values were considered statistically significant at *p* < 0.05. **(C)** Gating scheme for identification of CD123 expression on major lymphocyte and monocyte subsets in the peripheral blood. **(D)** Analysis of CD123 expression on major lymphocyte and monocyte subsets in the peripheral blood from a CpG ODN injected SQM. *A*liquots of the whole blood were stained with antibodies to B cells (CD20+; **a**), T cells (CD3+; **b**), NK cells (CD3-CD16+; **c**), and NKT cells (CD3+CD16+; **d**) to identify lymphocyte subpopulations and stained with antibodies to identify monocytes (CD14+; **e**) at day 0, 1, 3, and 7. Values on the Y-axis are % expressions of CD123 marker on lymphocyte and monocyte cell populations. Symbols representing significance are: ***p < 0.001; **p < 0.01; *p < 0.05; ns, *p* > 0.05.

**Figure 3:**
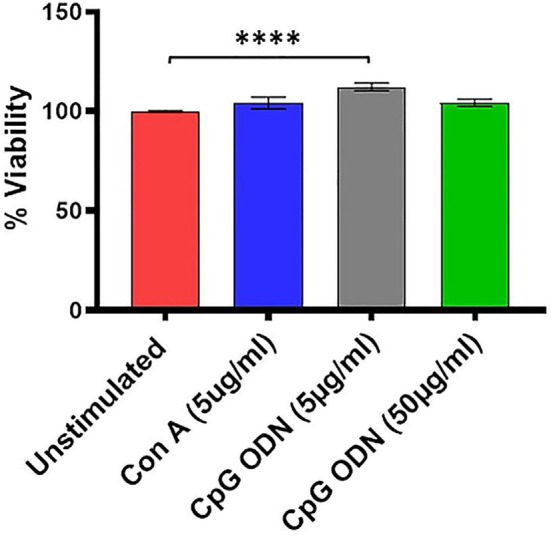
Viability assay. For viability of PBMCs we used a standard MTT proliferation dye reduction assay. PBMCs were cultured in triplicate wells at 1 × 10^5^/well in 96-well round-bottomed plates with 5 μg/ml and 50 μg/ml Class C CpG ODN or 5 μg/ml concanavalin A (Con A) for 96 h at 37°C. Proliferation responses were measured as optical density (OD) and data are presented as % viability over cells without stimulation. Symbols representing significance are: ****p < 0.0001.

**Figure 4:**
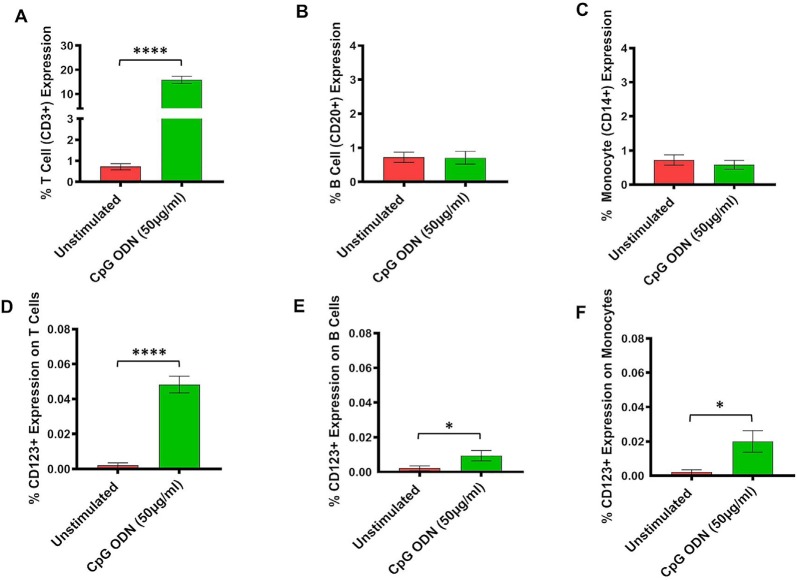
**(A–F)** Analysis of lymphocytes and monocytes expression in CpG ODN stimulated SQM PBMCs. PBMCs were isolated from the whole blood and aliquots of PBMCs were stimulated with 50 μg/ml CpG ODN for 30 h at 37°C. PBMCs were stained with antibodies to T cells (CD3+; **A)**, B cells (CD20+; **B)**, and monocytes (CD14+CD16−; **C)**. Values on the Y-axis **(A–C)** are % expression of lymphocytes and monocytes. Similarly, to identify CD123 expression on T cells (CD3+; **D**), B cells (CD20+; **E**), and monocytes (CD14+; **F**) PBMCs were stained with CD123. The samples were acquired on a FACS Celesta™ flow cytometer using DIVA software (BD) and analyzed using FlowJo (Tri Star, CA, USA). Values on the Y-axis **(D–F)** are % expressions of CD123 marker on lymphocyte and monocyte cell populations. Symbols representing significance are: ****p < 0.0001; *p < 0.05.

**Figure 5:**
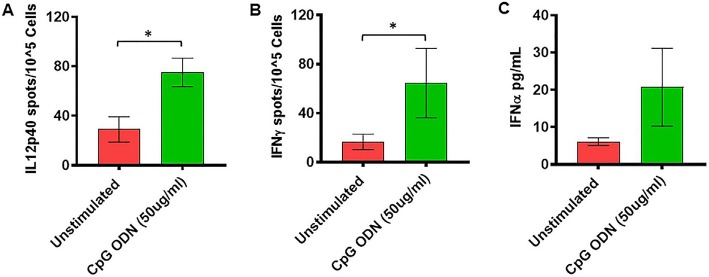
**(A,B)** IL2p40 and IFNγ cytokine ELISPOT assay. Freshly isolated PBMCs were either unstimulated (medium) or stimulated with CpG ODN (50 μg/ml) for 36 h at 37°C. The response to CpG ODN was evaluated by counting the total numbers of IL12p40 **(A)** and IFNγ **(B)** SFC adjusted to control medium as a background. **(C)** IFNα cytokine Luminex magnetic bead assay. In duplicate wells of the 96-well filter plate, PBMC cultures were stimulated with 50 μg/ml Class C CpG ODN for 36 h at 37°C. After incubation, 25 μl of cell-free supernatant was used for measurement of IFNα using Luminex technology (Bio-Plex 200 System) as described in material and methods. The minimum detectable concentration in pg/ml for IFNα (2.9) was used for considering positive responses. Symbols representing significance are: *p < 0.05.

For statistical analysis, samples were grouped according to treatment of the animals from which samples were obtained. Comparison analyses between samples collected post-treatment (Figures 1Ba–Bh, 2Da–De) were conducted using a one-way-ANOVA with repeated measures. Sphericity was not assumed, and Geisser–Greenhouse epsilon was used to correct the degrees of freedom. *Post hoc* comparison compared day 0 to days 1, 3, and 7 using a Dunnett’s test. Comparisons between groups without repeated sampling (Figures 2A,B, 3–5) were conducted using a one-way ANOVA when there were three or more groups and a one-tailed paired *t*-test when there were only two groups. A Dunnett’s test was done for *post hoc* analysis when there were three groups or more. Only differences with a probability less than 0.05 were considered to be significant. All statistical analyses were conducted using GraphPad Prism^®^ 8.0 (GraphPad Software, San Diego, CA, USA). Symbols representing significance are: ****p < 0.0001; ***p < 0.001; **p < 0.01; *p < 0.05; ns, *p* > 0.05.

## Results

### Expression of Major Lymphocyte and Monocyte Subsets in CpG ODN-Stimulated SQM Peripheral Blood

Peripheral blood samples from CpG ODN injected monkeys were analyzed by flow cytometry in order to enumerate various cell populations. To study kinetics in the expression of different lymphocyte and monocyte subsets, EDTA blood was collected prior to injection (baseline/day 0) and on day 1, day 3, and day 7 after CpG ODN administration. Phenotyping characterization of monocytes, T cells, B cells, NK cells, and NKT cells was performed using the logical gating strategy shown in Figure 1A. Results were analyzed using a one-way repeated measures analysis of variance. *Post hoc* analyses were conducted when appropriate using Dunnett’s test to compare day 0 to days 1, 3, and 7. Eta squared effect sizes were calculated to estimate the magnitude of the effect.

Absolute numbers of lymphocytes were directly obtained from Advia (Siemens Healthcare Diagnostics, Tarrytown, NY, USA). We observed a significant overall effect of days on the absolute number of lymphocytes (*F*_(2.16,10.1)_ = 33.97, *p* < 0.0001, *η*^2^ = 0.53; Figure 1Ba). *Post hoc* analysis indicated that there was a significant peak in lymphocytes expression on day 3 that remained significantly different on day 7 (Day 0 vs. Day 3, *p* < 0.001; Day 0 vs. Day 7, *p* < 0.05; Figure 1Ba). Subsequently, the lymphocyte subset assessment was performed by flow cytometry. The number of B cells (CD20+) decreased significantly across the study days (*F*_(1,3,11)_ = 15.42, *p* < 0.01, *η*^2^ = 0.51), with days 3 and 7 significantly lower than day 0 (Day 0 vs. Day 3, *p* < 0.01; Day 0 vs. Day 7, *p* < 0.01; Figure 1Bb). The number of T cells (CD3+CD20−) also declined significantly across study days (*F*_(2.5,19.9)_ = 40.02, *p* < 0.0001, *η*^2^ = 0.30), with days 3 and 7 significantly lower than day 0 (Day 0 vs. Day 3, *p* < 0.0001; Day 0 vs. Day 7, *p* < 0.0001; Figure 1Bc). We also observed a significant decrease in the absolute numbers of NKT cells (CD3+CD16+) across study days (*F*_(2.17,17.4)_ = 36.28, *p* < 0.0001, *η*^2^ = 0.23), with days 3 and 7 being significantly lower than day 0 (Dunnett’s *post hoc* test, Day 0 vs. Day 3, *p* < 0.0001; Day 0 vs. Day 7, *p* < 0.001; Figure 1Be). NK cells (CD3-CD16+) did not show any significant changes across study days (Figure 1Bd). Furthermore, a significant overall effect of days on the absolute numbers of monocytes obtained from Advia was present after CpG ODN administration (*F*_(1.3,10.1)_ = 25.99, *p* < 0.0001, *η*^2^ = 0.61; Figure 1Bf). *Post hoc* analysis revealed a significant peak in the numbers of monocytes on day 3 (Day 0 vs. Day 3, *p* < 0.01; Figure 1Bf). The absolute numbers of two subsets of the monocyte population were further evaluated by flow cytometry. Monocytes (CD14+CD16−) showed a significant day’s effect (*F*_(2,17.1)_ = 12.44, *p* < 0.001, *η*^2^ = 0.40), with day 3 significantly decreased compared to day 0 (Day 0 vs. Day 3, *p* < 0.001; Figure 1Bg). In addition, monocytes (CD14+CD16+) also showed a significant change across days (*F*_(2.02,16.2)_ = 6.52, *p* < 0.01, *η*^2^ = 0.22) with day 7 significantly lower than day 0 (Day 0 vs. Day 7, *p* < 0.05 ; Figure 1Bh).

In the next experiment, we positively identified plasmacytoid dendritic cells (pDCs) as Lin1-HLA DR+ population expressing CD123 marker (Figure 2A). We believe this is the first report describing pDC population in SQM species using a cross-reactive CD123 monoclonal antibody (Clone 7G3). We also tested various antibodies to identify CD11c+ cells (mDC). Unfortunately, none of the selected antibody clones presented cross-reactivity with SQMs. Furthermore, no significant differences in Lin-HLDR+ or Lin-CD123+ (pDC) expressing cells were observed between our two CpG ODN dose groups (doses of 5 μg/ml and 50 μg/ml; *in vitro*) and medium-only control group (Figure 2B). The gating strategy for our subsequent analysis of CD123 expression on major lymphocyte and monocyte subset populations is shown in Figure 2C. The expression of the CD123 marker is presented as a percentage of the parent population. There was significantly less CD123 expression on B cells (CD20+) across the study days (*F*_(2.16,17.24)_ = 4.59, *p* < 0.05, *η*^2^ = 0.29), with days 1 and 3 expressing significantly lower amounts of CD123 compared to day 0 (Day 0 vs. Day 1, *p* < 0.05; Day 0 vs. Day 3, *p* < 0.05; Figure 2Da). The amount of CD123 expressed on T cells (CD3+) also showed a significant change across study days (*F*_(1.6,13.2)_ = 6.88, *p* < 0.05, *η*^2^ = 0.26), with significantly lower expression on days 1 and 3 compared to day 0 (Day 0 vs. Day 1, *p* < 0.01; Day 0 vs. Day 3, *p* < 0.05; Figure 2Db). The amount of CD123 expressed on NK cells (CD3-CD16+) shows a significant effect of days across the study (*F*_(1.2,10.0)_ = 9.61, *p* < 0.01, *η*^2^ = 0.37), with expression significantly lower on days 1 and 3 compared to day 0 (Day 0 vs. Day 1, *p* < 0.01; Day 0 vs. Day 3 *p* < 0.001; Figure 2Dc). CD123 expression on NKT cells (CD3+CD16+) showed a significant increase across study days (*F*_(1.9,15.8)_ = 13.6, *p* < 0.001, *η*^2^ = 0.44), with expression significantly higher on days 3 and 7 compared to day 0 (Day 0 vs. Day 3, *p* < 0.01; Day 0 vs. Day 7, *p* < 0.05; Figure 2Dd). CD123 expression on monocytes (CD14+) showed a significant difference across study days (*F*_(1.5,11.8)_ = 4.75, *p* < 0.05, *η*^2^ = 0.34), with expression on day 3 significantly less than day 0 (Day 0 vs. Day 3, *p* < 0.01; Figure 2De).

### Viability Assay

We further examined the viability as well as functional activity of PBMCs exposed to different concentrations of Class C CpG ODN. PBMCs isolated from SQMs were cultured in the presence or absence of CpG ODN (doses of 5 μg/ml or 50 μg/ml) and viability was measured using MTT assay. Mitogen Con A was used as positive control. The results were analyzed using a one-way ANOVA for repeated measures. Where appropriate, a Dunnett’s test was used to compare treatment groups to the medium-only sample. Eta squared was calculated to estimate the magnitude of the effect size.

There was a significant treatment effect (*F*_(1.9,45.1)_ = 7.08, *p* < 0.01, *η*^2^ = 0.17) with *post hoc* analysis revealing significantly increased percent viability in PBMCs stimulated with 5 μg/ml of CpG ODN compared to medium-only control cells (*p* < 0.0001; Figure 3). No significant differences were detected between the medium group and either the Con A or the CpG ODN (50 μg/ml) groups, further confirming the viability of PBMCs (Figure 3).

### Expression of Major Lymphocyte and Monocyte Subsets in CpG ODN-Stimulated SQM PBMCs

Subsequently, the assessment of CpG ODN immunostimulatory activity was examined in PBMCs isolated from SQMs and incubated in the presence or absence of a CpG ODN (50 μg/ml, predetermined safe dose) for 30–32 h. PBMCs were further subjected to flow cytometry to determine expression frequency of T cells, B cells, and monocytes, as well as the activation status of CD123 expression on those particular cell populations. The results were analyzed using a one-tailed paired *t-*test to compare the background medium results to those treated with CpG ODN. Hedge’s g statistic was calculated to estimate the magnitude of the effect size.

There was a significant increase in the percent of T cells (CD3+) expression (*t* = 14.7, *df* = 12, *p* < 0.0001, *g* = 5.46; Figure 4A). However, there were no differences in expression frequencies of B cells (CD20+; Figure 4B) and monocytes (CD14+; Figure 4C) between the CpG ODN group and the medium control group. Moreover, significantly increased expressions of CD123+ were detected on T cells (CD3+; *t* = 9.87, *df* = 12, *p* < 0.0001, *g* = 3.26; Figure 4D), B cells (CD20+; *t* = 2.27, *df* = 12, *p* < 0.05, *g* = 0.89; Figure 4E), and monocytes (CD14+; *t* = 2.61, *df* = 12, *p* < 0.05, *g* = 1.05; Figure 4F) in response to CpG ODN stimulation when compared to the medium only group.

### Measurement of Cytokines by ELISPOT and Luminex Magnetic Bead Assay

Freshly isolated PBMCs were either unstimulated (medium) or stimulated with nontoxic doses of class C CpG ODN (5 μg/ml and 50 μg/ml). The numbers of IFNγ- and IL12p40-producing cells were determined by ELISPOT assay. Results were analyzed using a one-tailed paired *t*-test. Hedge’s g statistic was calculated to estimate the magnitude of the effect size.

As shown in Figure 5A, significantly higher numbers of IL12p40-producing cells were detected in response to stimulation with 50 μg/ml of CpG ODN (*t* = 2.51, *df* = 10, *p* < 0.05, *g* = 1.29) compared to unstimulated PBMCs. Similarly, the numbers of IFNγ-producing cells were significantly higher after stimulation with 50 μg/ml of CpG ODN compared to medium only (*t* = 2.1, *df* = 9, *p* < 0.05, *g* = 0.71; Figure 5B). No significant differences were observed in response to 5 μg/ml CpG ODN stimulation of PBMCs compared to the medium control group (data not shown). In addition, the ability of CpG ODN to induce IFNα from SQM PBMC cultures was evaluated in cell-free supernatant using Luminex technology. There were no statistical differences between medium and IFNα levels (Figure 5C) in CpG ODN (50 μg/ml)-treated cultures. Although the mean levels are higher in the CpG ODN-treated samples, the variance in the values overshadows these differences. Further research is needed to understand the differences between samples that responded to CpG ODN treatment and those that did not.

## Discussion

When eliciting a desirable immune response against human disease, modeling therapeutic approaches in non-human primates (NHPs) is advantageous because of the highly conserved innate immune system among primates (Messaoudi et al., 2011). One of these highly conserved immunological mechanisms in humans and in NHPs is the recognition of unmethylated cytosine-phosphate-guanine (CpG) DNA that is commonly found in the genomes of prokaryotes, while being underrepresented in those of eukaryotes. CpG oligodeoxynucleotides (CpG ODNs) trigger cells that express TLR9 to mount an innate immune response (Krieg, 2002; Crack and Bray, 2007; Vollmer and Krieg, 2009). Our initial findings from AD mouse models provide the first *in vivo* evidence that stimulation of innate immunity *via* TLR9 with CpG ODN appears to reduce behavioral deficits and restrict all pathological hallmarks of AD, including amyloid plaques, tau pathology, and CAA without associated toxicity (Scholtzova et al., 2009, 2014, 2017). Due to interspecies differences in TLR9 expression, caution needs to be taken when attempting to apply data from rodents to primates. Rodents exhibit different immune response patterns to CpG ODN than that observed in NHPs and humans, since murine TLR9 is expressed in a broader range of immune cells, whereas primate TLR9 expression is restricted to B lymphocytes and plasmacytoid dendritic cells (pDCs; Krieg, 2006, 2012; Scheiermann and Klinman, 2014). Furthermore, function and expression of TLRs can be affected by immunosenescence in the elderly population (Renshaw et al., 2002; Fiala et al., 2007). Age-associated changes in immune cell functions have been recognized in earlier reports, including our own work (Fiala et al., 2005; Panda et al., 2010; Nehete et al., 2013; Hou et al., 2019). Although there have been several studies using NHPs to evaluate the efficacy of CpG ODN for various clinical indications, there are no published data of CpG ODN having been used effectively for preventive or therapeutic interventions in neurodegenerative disorders, such as AD or CAA, in either NHPs or humans (Hartmann et al., 2000; Verthelyi et al., 2002; Verthelyi and Klinman, 2003; Teleshova et al., 2006; Verthelyi, 2006).

*Saimiri boliviensis boliviensis* (SQMs) were utilized in the present study due to their unique characteristic of naturally occurring amyloid-related pathology. An important feature of Aβ depositions in SQMs is their species-specific propensity to CAA (Elfenbein et al., 2007; Heuer et al., 2012, 2017; Rosen et al., 2016; Jäkel et al., 2017; Devinsky et al., 2018). CAA is almost universally present in AD. However, it can also occur independently (Jellinger and Attems, 2005; Charidimou et al., 2017). The severity of CAA is an independent risk factor for dementia. While there are no effective treatments for AD or CAA (Drummond et al., 2018; Lane et al., 2018; Smith, 2018; Weber et al., 2018), immunomodulatory approaches are showing meaningful promise as therapeutic interventions for AD (Herline et al., 2018; Long and Holtzman, 2019). Furthermore, current immunotherapeutic approaches in human clinical trials are associated with adverse occurrence of amyloid related imaging abnormalities (ARIA). These complications are linked to rapid clearance of CAA, resulting in blood-barrier breakdown and excessive neuroinflammation (Wisniewski and Goñi, 2015; Herline et al., 2018; Elmaleh et al., 2019). Hence, the SQM represents an essential model for testing immunomodulatory approaches for neurodegenerative disorders due to its accurate mimicry of human CAA that could potentially lead to development of ARIA, and by having an immune system much more proximate to humans (Heuer et al., 2012, 2017; Drummond and Wisniewski, 2017). The interventions described here represent the first study using elderly SQM with established brain pathology as a model to assess the magnitude of immune responses to Class C CpG ODN. The TLR9 agonist Class C CpG ODN is currently being explored in humans for a variety of disease indications, and has shown favorable safety profiles (Vollmer and Krieg, 2009; Gosu et al., 2012; Krieg, 2012; Wang et al., 2016; Ribas et al., 2018).

Class C CpG ODN containing both features of Class A and B was used to assess immune response characteristics, including phenotypes and frequency of lymphocyte/monocyte subsets, PBMC viability, and functionality in SQM peripheral blood and PBMCs (Vollmer et al., 2004; Martinson et al., 2007). Expressions of major lymphocyte and monocyte subsets were first examined in whole blood collected prior to s.c. CpG ODN injection and at multiple intervals post-CpG ODN injection. A significant peak in the absolute number of total circulating lymphocyte and monocyte populations was detected on day 3 after CpG ODN administration as assessed by hematology. In contrast, flow cytometry enumeration of major lymphocyte subsets including B cells (CD20+), T cells (CD3+), and NKT cells (CD3+CD16+), revealed significant decreases in the absolute numbers across study days, with days 3 and 7 being significantly lower than baseline taken just prior to injection. In addition, classical monocyte population expressing CD14+ cell surface receptor (CD14+CD16−) showed significant change across days, with 3 days post injection being significantly lower than baseline. Non-classical monocytes co-expressing CD16+ (CD14+D16+) demonstrated significant decline 7 days post injection. These results might likely reflect transient redistribution of cells out of peripheral blood. It is possible that identified declines of specific lymphocyte and monocytes subsets across days are being mediated by immune cell migration to specific sites including peripheral organs (e.g., lymph nodes, spleen, liver), site of CpG ODN administration, and various pathology sites (e.g., tumors, sites of neurodegeneration/brain pathology; Krieg et al., 2004; Haining et al., 2008; Murad and Clay, 2009; Kumar et al., 2015; Ribas et al., 2018). Our earlier studies showed that peripheral administration of CpG ODN in experimental mouse models of AD was associated with transient recruitment of immunoregulatory cells, including monocyte-macrophages, to cerebral site of amyloid plaque pathology (Scholtzova et al., 2009, 2014, 2017). However, manifestation of target organ toxicity represented as the occurrence of leukocyte infiltrates within the major organs of deposition has not been observed in our initial mouse reports, further supporting the safety of this immunomodulatory approach (Scholtzova et al., 2014, 2017). In addition, the present findings should be interpreted with caution, because only selected cross-reactive reagents with specific cell subpopulations were included in this study (de Souza et al., 2017). It is also important to note that the small size of SQMs limits the volume of blood that can be collected at any time point.

Two major populations of dendritic cells (DCs), myeloid and plasmacytoid, can be isolated from human peripheral blood (PBMCs), and are distinguished by differential expression of the cell surface markers CD11c and CD123. These two populations of DCs are also different in their expression of TLRs, which are involved in their activation (Krug et al., 2001; Rothenfusser et al., 2002). While the CD11c antibody clone did not cross-react with SQM, we demonstrated CD123 cross reactivity in SQM for the first time. Thus, plasmacytoid dendritic cells (pDCs) were positively identified as Lin-HLA DR+ population expressing the CD123 marker. In the next experiment, peripheral blood samples collected from CpG ODN-injected monkeys were analyzed by flow cytometry to study the kinetics of CD123 expression, also known as IL3 receptor α chain (IL3Rα), on the surface of major lymphocyte and monocyte subset populations. Decreased expression of CD123 on B cells (CD20+), T cells (CD3+), NK cells (CD3-CD16+), and monocytes (CD14+) was observed on days 1 and 3 after s.c CpG ODN injection, when compared to baseline. The drop was transient as baseline counts returned by day 7. Even though we were not able to perform a more comprehensive evaluation of cell activation/maturation markers (functional characteristics) due to insufficient volume of blood and restricted accessibility of cross-reactive reagents, the possibility remains that the observed decline of the CD123 marker could be related to an ongoing activation process. It has been shown that CD123 expression decreases with activation (Rothenfusser et al., 2002). Another potential explanation is that these reductions in the CD123 co-expressing marker may be reflecting migration of CD123+ parent cell population to peripheral tissue.

To better study the immunomodulatory capacity of CpG ODN effects, we confirmed the viability of PBMCs exposed to different concentrations of Class C CpG ODN. Further evidence of CpG ODN activity was obtained from SQM PBMCs assayed by flow cytometry to evaluate cell surface markers after incubation with CpG ODN. Even though no differences in expression frequencies of B cells and monocytes were detected between the CpG ODN-stimulated PBMCs and the unstimulated control group, there was a significant increase in the percentage of T cells expression. We additionally evaluated the percentage of CD123 marker expression on the surface of major lymphocyte and monocyte subsets after PBMC stimulation with CpG ODN. Here, we provide an evidence that CpG ODN can increase the percentage of CD123+ on T and B lymphocyte subsets, as well as monocyte population when compared to unstimulated cells. It is possible that part of the effect we observed here was related to enhanced IL3R signaling that has been shown to promote proliferation and cell viability (Macardle et al., 1996; Teleshova et al., 2006; Wittwer et al., 2017). The disparity between the CD123 co-expression levels observed in CpG ODN-stimulated peripheral blood (s.c injection) and in CpG ODN-stimulated SQM PBMCs may be caused by varying differentiation stages of specific analyzed cell subsets at the time of stimulation with CpG ODN. Others have reported that identification of specific cell subset stimulatory activity within PBMCs is limited to specific time points (Rothenfusser et al., 2002; Krug et al., 2003; Teleshova et al., 2006; Gujer et al., 2011). Hence, stimulatory activity may be restricted to time points that were not examined due to a limited number of SQM PBMCs. Moreover, data presented here were evaluated using total PBMCs, and responses specific to individual lymphocyte subpopulations would need to be addressed independently to understand CpG ODN’s role on individual cell types.

Subsequent validation of class C CpG ODN’s immunostimulatory potential was obtained in SQM PBMCs by demonstrating increased production of selected cytokines. Using an ELISPOT assay, PBMCs were tested for functionality after incubation with two doses of CpG ODN. While no significant differences were observed for the numbers of IL12- and IFNγ-producing cells in response to stimulation with a low dose of CpG ODN, there was a significant increase seen in the numbers of cells producing cytokines in response to stimulation with our higher dose of CpG ODN compared to unstimulated cells. We believe that as aging occurs, higher doses of CpG ODN may be required to detect a significant magnitude of immune response as implied by our previously published reports that indicated less potent responses to several mitogens in elderly SQMs (Nehete et al., 2013). These functional activity data are further supported by the previously reported ability of class C CpG ODN to stimulate substantial IL12 and IFNγ production in primates (Duramad et al., 2003; Jurk et al., 2004; Teleshova et al., 2004). Immune cell subsets including pDCs, mDCs, B cells, and monocytes have been implicated in IL12 induction (Poeck et al., 2004; Bekeredjian-Ding et al., 2006; Hanagata, 2012). IFNγ production after CpG ODN stimulation is mainly facilitated by T cells and NK cells (Yi et al., 1996; Yin et al., 2012). In addition, the ability of CpG ODN to induce IFNα from SQM PBMC cultures was evaluated in cell-free supernatant using Luminex technology. It has been demonstrated that pDC are the main IFNα-producing cells after CpG ODN stimulation in humans and monkeys (Marshall et al., 2005; Martinson et al., 2007). Although, as expected, increased concentration of IFNα was detected in PBMC culture treated with CpG ODN compared to medium-treated culture, the levels did not differ significantly. Our less robust IFNα responses are consistent with previous reports of reduced type I IFN production in aged subjects (Panda et al., 2010; Asquith et al., 2012).

In conclusion, we confirmed the effectiveness of Class C CpG ODN to elicit an immune response in an aged population whose immune system might be compromised. The specific cytokine profiles described here closely mirrored CpG ODN-induced immunostimulatory properties observed in earlier human studies, further supporting the use of the SQM model (Duramad et al., 2003; Jurk et al., 2004; Marshall et al., 2005; Martinson et al., 2007). Additionally, the present findings provide the first evidence demonstrating the cross-reactivity of the CD123 marker with SQM lymphocytes. Whether CpG ODN can safely harness innate immunity to rescue the CAA pathology in elderly monkeys is being assessed in our ongoing CpG ODN chronic treatment study. Although further analysis with sensitive SQM-reactive reagents is needed, the study described here provides essential information regarding immune response patterns of CpG ODN in this proximate NHP model, enabling future translational research for various indications to reach potential clinical applicability of CpG ODN.

## Data Availability Statement

The datasets generated for this study are available on request to the corresponding author.

## Ethics Statement

The animal study was reviewed and approved by (1) the NYU—Institutional Animal Care and Use Committee and (2) The University of Texas, MD Anderson Cancer Center—Institutional Animal Care and Use Committee. This research was conducted at the AAALAC-I accredited Michale E. Keeling Center for Comparative Medicine and Research, UT MD Anderson Cancer Center, Bastrop, TX, USA (Common SQMs 03-09-02781). All animal experiments were carried out according to the provisions of the Animal Welfare Act, PHS Animal Welfare Policy, and the principles of the NIH Guide for the Care and Use of Laboratory Animals (National Research Council, 2011). Blood sampling volumes were approved by the IACUC and the clinical veterinarian, and the monkeys were healthy throughout the study. All procedures were approved by the Institutional Animal Care and Use Committee at the UT MD Anderson Cancer Center.

## Author Contributions

PN designed and supervised experiments, analyzed and interpreted data, and wrote the manuscript. LW performed statistical analyses and contributed to manuscript preparation. The head of the subcontract at the Michale E. Keeling Center for Comparative Medicine and Research, UT MD Anderson Cancer Center, Bastrop, Texas. SC performed experiments (Flow Cytometry). BN performed experiments (ELISPOT and LUMINEX based cytokine assays, Viability MTT assay). AP conducted background literature search and contributed to manuscript preparation. MR contributed to manuscript preparation. TW contributed to experiment development, data interpretation, and manuscript preparation. HS conceived and supervised the research, contributed to experiment development and data interpretation, and wrote the manuscript.

## Conflict of Interest

The authors declare that the research was conducted in the absence of any commercial or financial relationships that could be construed as a potential conflict of interest.

## Footnotes

^1^www.nhpreagents.org
